# Associations Between Periviable and Preterm Birth and Severe Maternal Morbidity and Mortality

**DOI:** 10.1097/og9.0000000000000085

**Published:** 2025-06-05

**Authors:** Heather Czarny, Stefany Hernandez, Isabella Toledo, Elizabeth Kelly, William Moravec, Carri Warshak, Emily Defranco, Robert Rossi

**Affiliations:** Department of Obstetrics and Gynecology, University of Cincinnati College of Medicine, Cincinnati, Ohio; the Department of Obstetrics and Gynecology, Indiana University School of Medicine, Indianapolis, Indiana; and the Department of Obstetrics and Gynecology, University of Kentucky College of Medicine, Lexington, Kentucky.

## Abstract

**OBJECTIVE:**

To evaluate the risk of severe maternal morbidity (SMM) and mortality associated with periviable or preterm birth.

**METHODS:**

This population-based retrospective cohort study analyzed U.S. delivery hospitalizations between the fourth quarters of 2015 and 2021 by using the National Inpatient Sample database. The primary outcome was SMM and mortality risk by gestational age (20–25 weeks vs 26–36 weeks vs 37–42 weeks). Multivariable logistic regression analysis was used to assess SMM risk. Secondary outcomes included organ system–based SMM indicators and subgroup analyses by 3-week gestational age intervals (eg, 20–22 weeks, 23–25 weeks). Population attributable fraction (PAF) estimates were calculated to determine the proportion of SMM due to periviable and preterm birth.

**RESULTS:**

Among an estimated 22,208,488 weighted births, 0.6% were periviable (20–25 weeks), 9.4% were preterm (26–36 weeks), and 90.0% were term (37–42 weeks). Severe maternal morbidity and mortality rates were highest in individuals with periviable births (4.5%, adjusted relative risk [aRR] 6.8 95% CI, 6.4–7.3, 98/100,000 delivery hospitalizations; and aRR 26.4, 95% CI, 16.7–41.9, respectively) and preterm births (2.9%, aRR 3.6, 95% CI, 3.5–3.7, 31/100,000 delivery hospitalizations; and aRR 8.7, 95% CI, 6.4–11.7, respectively) compared with term births (0.5%, 3/100,000 delivery hospitalizations). The elevated risk for SMM remained when limited to spontaneous live births in individuals without chronic comorbidities associated with iatrogenic preterm delivery (periviable: aRR 3.8, 95% CI, 3.3–4.5; preterm: aRR 1.8, 95% CI, 1.7–2.0). Individuals with periviable births had the highest risk of cardiovascular, renal, respiratory, sepsis, shock, and hemorrhagic SMM events, and those with preterm births had the highest risk of eclampsia. The PAF for SMM and maternal death was highest in individuals with preterm births (26.3%, 95% CI, 25.6–27.1; and 48.1%, 95% CI, 39.9–55.2, respectively).

**CONCLUSION:**

Periviable and preterm birth is associated with an increased risk of SMM and mortality, emphasizing the maternal health burden associated with early deliveries and the need for coordinated maternal–fetal risk-appropriate care.

## Abstract 2

Periviable and preterm delivery is associated with an increased risk of severe maternal morbidity and mortality compared with term delivery, underscoring the need for coordinated maternal–fetal regionalization of care.

Preterm birth is a significant global burden, affecting nearly 10% of births worldwide^1^ and accounting for approximately 18% of deaths among children younger than age 5 years.^2^ The United States exhibits the highest preterm birth rate among developed nations, echoing the global prevalence.^1,3^ Two in three preterm births are spontaneous; the remainder are indicated early deliveries due to complications such as preeclampsia or fetal growth restriction.^4,5^

There is growing evidence that preterm birth is associated with increased risk for severe maternal morbidity (SMM), with the highest risk at the extremes of prematurity.^6–9^ Prior studies have evaluated the dual burden of preterm birth and SMM, estimating that preterm birth and SMM occur in 1 of every 270 births,^10^ with the highest incidence of SMM among births occurring at less than 28 weeks of gestation.^9^ However, although these studies provide some data on risk, they are not necessarily generalizable to the entire U.S. population because there is significant variation in preterm birth and SMM rates geographically.^11,12^ Further, there is limited evidence that explores the association between SMM and mortality risk and gestational age at delivery, viability, delivery indication, and the character of the SMM episode in the preterm period.^9,10^

The objective of this study was to characterize composite SMM as well as each individual SMM indicator, potentially preventable SMM, and maternal mortality risk among individuals with periviable (20–25 weeks of gestation), preterm (26–36 weeks), and term (37–42 weeks) deliveries.

## METHODS

This was a population-based, retrospective cohort study of delivery hospitalizations in the U.S. that used the National Inpatient Sample (NIS) database from the Agency for Healthcare Research and Quality's Healthcare Cost and Utilization Project.^13^ The NIS is the largest publicly available all-payer inpatient health care database that approximates a 20% stratified sample of all U.S. community hospitals and represents 97% of the U.S. population, with data contributed from Healthcare Cost and Utilization Project partners within participating states.^13^ Analysis of NIS data is not considered human research and was exempt from review by our institution's IRB.

Inclusion criteria included female-identified individuals between age 10 and 57 years with an associated delivery hospitalization at or after 20 weeks of gestation between the fourth quarters of 2015 and 2021. Individuals for whom gestational age at delivery was missing and those transferred to an acute care hospital were excluded from the analysis. Delivery hospitalization was constructed using a previously described enhanced identification method with modifications using the International Classification of Diseases, Tenth Revision, Clinical Modification (ICD-10-CM) and Procedure Coding System (ICD-10-PCS), Medicare Severity-Diagnosis Related Groups, and ICD procedure codes.^14–16^ Gestational age at the time of delivery was determined by ICD-10-CM codes.^17^ Admissions outside of delivery hospitalization were not included in the analysis. STROBE (Strengthening the Reporting of Observational Studies in Epidemiology) and RECORD (REporting of studies Conducted using Observational Routinely-Collected Data) guidelines were followed for this study.^18^

Severe maternal morbidity events were identified using individual SMM indicator ICD-10 codes as published by the Centers for Disease Control and Prevention and the Alliance for Innovation on Maternal Health (Appendix 1, available online at http://links.lww.com/AOG/E152).^15,16^ Severe maternal morbidity indicated solely by blood transfusion without other SMM indicator codes may overestimate SMM; therefore, it was not included in the primary definition of SMM.^19,20^ Blood transfusion alone or in combination with other SMM indicators was included in subgroup analyses and is referred to as transfusion SMM. Maternal death was identified using coded discharge disposition within the NIS data files.

Potentially preventable SMM is a previously described method to define hospital-acquired SMM.^21^ This definition excludes conditions thought to be nonpreventable, such as amniotic fluid embolism, and SMM that is present on admission. These outcomes were identified within the NIS database through ICD-10 codes and excluded SMM listed as the first diagnosis, as this represents the principal diagnosis for admission and is presumed to be present on admission.^22^

Severe maternal morbidity indicators were analyzed individually and also were categorized by affected organ system for subgroup analyses due to the infrequency of certain individual events. Categories included: cardiovascular SMM (acute myocardial infarction; aneurysm; cardiac arrest or ventricular fibrillation; conversion of cardiac rhythm; pulmonary edema or acute heart failure; and puerperal cerebrovascular disorders), hemorrhage SMM (transfusion and disseminated intravascular coagulation), renal SMM (acute renal failure), respiratory SMM (adult respiratory distress syndrome, severe anesthesia complication, temporary tracheostomy, and ventilation), thromboembolic SMM (amniotic fluid embolism, sickle cell disease with crisis, and air and thrombotic embolism), along with shock, sepsis, eclampsia, and hysterectomy.

Additional covariates in our analysis included socioeconomic and demographic maternal characteristics available through the NIS database, such as age, race or ethnicity, payer, hospital location, and teaching status. Racial disparities in preterm birth and SMM are well-documented in the U.S.^5,10,11^ These disparities are not attributable to race as a biological construct, but rather reflect the effects of structural racism, discrimination, and socioeconomic inequities that affect access to and quality of care.^23^ Because the NIS data set lacks granular measures of these underlying social determinants, we included race and ethnicity, along with insurance status and hospital characteristics, to partially account for these confounding influences and improve the accuracy of our risk adjustment. Race and ethnicity are collected by Healthcare Cost and Utilization Project partner hospitals.^13^ Details regarding how each institution collects these data are not available through the NIS.^13^ Medical comorbidities, substance use, pregnancy-related diagnoses, and obstetric outcomes were identified through ICD-10-CM codes (Appendix 2, available online at http://links.lww.com/AOG/E152).

Descriptive statistics were used to analyze differences in the prevalence of baseline characteristics, maternal comorbidities, and pregnancy outcomes by gestational age at delivery. Gestational age at delivery was categorized into three epochs: periviable (20–25 weeks), preterm (26–36 weeks), and term (37–42 weeks). The periviable gestational age group was chosen to encompass the spectrum of neonatal survival and intervention rates ranging from 0% to nearly 100%.^24^ Rates of SMM, potentially preventable SMM, individual SMM indicators, SMM by organ system category, and maternal death were compared by gestational age at delivery epoch. A composite of adverse obstetric outcomes (preeclampsia with severe features, intrauterine fetal death, fetal growth restriction, and placental abruption) also was assessed. Lastly, subgroup analyses were performed for SMM, SMM by organ system classification, and maternal death by 3-week stratified gestational age epochs (eg, 20–22 weeks, 23–25 weeks).

We performed multivariable logistic regression analysis to estimate the risk for the primary and secondary outcomes by gestational age at delivery using a backward selection process of biologically plausible characteristics and those with significant differences between exposure cohorts and *P*<.05.^25^ The variables selected for the adjusted model included advanced maternal age, race and ethnicity, Medicaid or Medicare insurance, hospital location and teaching status, psychiatric disease, chronic cardiac disease, chronic hypertension, severe preeclampsia, pregestational diabetes, twins or higher-order multiple gestation, anemia, and prior cesarean delivery. Sensitivity analyses also were performed among individuals with live births for the primary and secondary outcomes to estimate risk for different groups given the inherent differences in SMM risk profile based on the delivery indication.^26–32^ The groups included live births only, live births delivered vaginally, singleton live births, medically indicated live births, spontaneous live births, live births among individuals without chronic conditions often necessitating preterm delivery (chronic cardiovascular, renal, or pulmonary disease, chronic hypertension, and pregestational diabetes), and live births among those without chronic conditions who delivered spontaneously. We broadly classified birth as iatrogenic (labor induction, fetal growth restriction, hypertensive disorders of pregnancy) or spontaneous (preterm labor, preterm prelabor rupture of membranes).

To estimate the theoretical possibility of SMM prevention as a proportion of the total SMM burden among those with periviable or preterm birth, the adjusted population attributable fraction (aPAF) was calculated.^33^ STATA 15.1 software was used to perform statistical analysis. *P<*.05% and 95% CI not inclusive of the null value of 1.0 indicated statistical significance.

## RESULTS

**Fig. 1.**
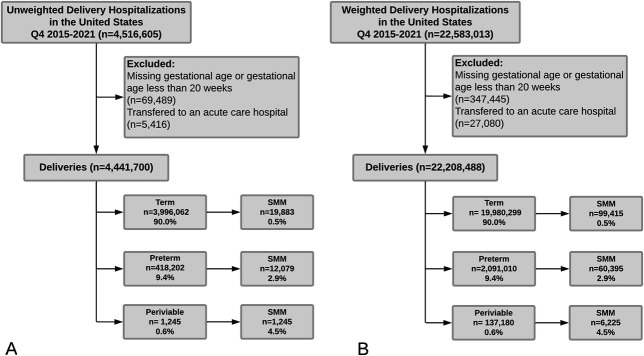
**A.** Unweighted study population representing 20% stratified sample of delivery hospitalizations within the National Inpatient Sample. **B.** Weighted study population approximating national delivery hospitalizations. SMM, severe maternal morbidity.

There were 4,516,605 delivery hospitalizations within the NIS database, with a weighted estimate of 22,583,013 delivery hospitalizations. After excluding individuals transferred to acute care hospitals, those with missing gestational age at delivery hospitalization, and those with delivery hospitalization before 20 weeks of gestation, 22,208,488 weighted delivery hospitalizations were included in this analysis. Of these, 0.6% deliveries occurred in the periviable period, 9.4% in the preterm period, and 90.0% in the term period (Fig. 1). Individuals with periviable or preterm deliveries were more likely to identify as Black, be aged 35 years or older, have Medicaid or Medicare insurance, and deliver at an urban teaching hospital compared with those with term deliveries (Table 1). Individuals with deliveries before term were also more likely to use tobacco or other substances, have chronic comorbidities (hypertension, pregestational diabetes, and cardiovascular, renal, respiratory, and psychiatric diseases), have adverse obstetric outcomes (preeclampsia with severe features, intrauterine fetal death, fetal growth restriction, chorioamnionitis) or multifetal gestations, and to have premature rupture of membranes or undergo cesarean delivery (Table 1).

*Czarny. SMM With Periviable and Preterm Birth. O&G Open 2025*.

**Table 1.** Baseline Characteristics by Gestational Age at Delivery*

Characteristic	Gestational Age Category (wk of Gestation)		
	Periviable (20–25) (n=137,180)	Preterm (26–36) (n=2,091,009)	Term (37–42) (n=19,980,299)
Race and ethnicity			
American Indian	1,080 (0.8)	18,175 (0.9)	136,510 (0.7)
Asian or Pacific Islander	5,590 (4.1)	104,730 (5.0)	1,201,189 (6.0)
Black	42,525 (31.0)	410,250 (19.6)	2,727,110 (13.6)
Hispanic	25,845 (18.8)	416,380 (19.9)	4,056,193 (20.3)
White	49,725 (36.3)	967,950 (46.3)	10,166,888 (50.9)
None of the above	6,295 (4.6)	88,130 (4.2)	871,010 (4.4)
Maternal age (y)	28.9±6.3	29.4±6.2	29.0±5.8
Advanced maternal age (35 y or older)	28,220 (20.6)	452,040 (21.6)	3,582,148 (17.9)
Medicaid or Medicare insurance	69,040 (50.3)	1,007,305 (48.2)	8,416,510 (42.1)
Hospital location and teaching status			
Rural	6,890 (5.0)	142,365 (6.8)	1,851,996 (9.3)
Urban nonteaching	17,080 (12.5)	351,055 (16.8)	4,050,687 (20.3)
Urban teaching	113,210 (82.5)	1,597,590 (76.4)	14,077,615 (70.5)
Obesity class III (BMI 40 or higher)	7,630 (5.6)	133,895 (6.4)	809,725 (4.1)
Tobacco use	10,710 (7.8)	164,975 (7.9)	951,689 (4.8)
Substance use†	13,975 (10.2)	209,775 (10.0)	1,134,839 (5.7)
Prior cesarean delivery	19,195 (14.0)	428,075 (20.5)	3,508,518 (17.6)
Anemia	23,790 (17.3)	386,330 (18.5)	3,028,454 (15.2)
Chronic hypertension	12,940 (9.4)	167,835 (8.0)	623,450 (3.1)
Chronic cardiac disease	1,795 (1.3)	23,685 (1.1)	99,620 (0.5)
Chronic renal disease	1,630 (1.2)	14,875 (0.7)	34,940 (1.7)
Chronic respiratory disease	9,620 (7.0)	149,580 (7.2)	1,066,279 (5.3)
Pregestational diabetes	4,750 (3.5)	95,590 (4.6)	230,885 (1.2)
Psychiatric diagnosis	16,985 (12.4)	257,310 (12.3)	1,615,644 (8.1)
Thyroid disorder	7,215 (5.3)	114,750 (5.5)	868,660 (4.3)
COVID-19	1,160 (0.8)	17,055 (0.8)	92,160 (0.5)
ART	900 (0.7)	9,980 (0.5)	37,175 (0.2)
Labor induced	16,630 (12.1)	310,760 (14.9)	4,592,482 (23.0)
Cesarean delivery	46,680 (34.0)	1,017,335 (48.7)	6,086,237 (30.5)
Multifetal gestation	14,415 (10.5)	224,295 (10.7)	152,060 (0.8)
Preeclampsia with severe features	12,660 (9.2)	414,015 (19.8)	390,095 (2.0)
Gestational diabetes	5,215 (3.8)	239,410 (11.4)	1,590,594 (8.0)
Fetal growth restriction	12,255 (8.9)	217,115 (10.4)	594,155 (3.0)
Intrauterine fetal death	56,055 (40.9)	56,535 (2.7)	36,715 (0.2)
Abruption	17,640 (12.9)	113,055 (5.4)	115,870 (0.6)
Chorioamnionitis	19,095 (13.9)	43,525 (2.1)	464,150 (2.3)
Placenta accreta spectrum	810 (0.6)	14,435 (0.7)	11,785 (0.1)
Premature rupture of membranes	44,275 (32.3)	516,875 (24.7)	1,368,574 (6.8)
Adverse obstetric outcome‡	81,880 (59.7)	696,375 (33.3)	1,105,864 (5.5)
Medically indicated delivery§	36,495 (26.6)	865,100 (41.4)	6,138,532 (30.7)
Spontaneous delivery||	81,370 (59.3)	1,107,095 (52.9)	1,187,014 (5.9)

BMI, body mass index; COVID-19, coronavirus disease 2019; ART, assisted reproductive technology.

Data are n (%) or mean±SD.

* All values differ significantly (*P*<.001). Counts included are weighted from the National Inpatient Sample.

† Includes alcohol, opioid, sedative, cocaine, cannabis, amphetamine, hallucinogen, a combination of, or unspecified drug use, abuse, or dependence.

‡ Includes severe preeclampsia, intrauterine fetal death, fetal growth restriction, and placental abruption.

§ Includes International Classification, Tenth Revision (ICD-10) diagnosis codes for fetal growth restriction, preeclampsia, and induction of labor.

|| Includes ICD-10 diagnosis codes for spontaneous preterm labor and delivery, preterm premature rupture of membranes, and premature rupture of membranes; excludes ICD-10 diagnosis codes for induction of labor.

For the primary outcome, the rates of SMM (4.5% vs 2.9% vs 0.5%) and transfusion SMM (7.3% vs 4.9% vs 1.4%) were significantly higher among those who delivered in the periviable and preterm period compared with term, respectively (*P*<.001) (Table 2). The adjusted relative risks (aRRs) for SMM and transfusion SMM were significantly increased among patients who delivered in the periviable (aRR 6.8, 95% CI, 6.4–7.3; and aRR 4.2, 95% CI, 3.9–4.4) and preterm (aRR 3.6, 95% CI, 3.4–3.7; and aRR 2.2, 95% CI, 2.2–2.3) periods compared with term. Potentially preventable SMM followed a similar pattern of SMM risk, but to a lesser degree (periviable: 3.6%, aRR 5.0, 95% CI, 4.6–5.5; preterm: 1.9%, aRR 2.3, 95% CI, 2.2–2.3). The rate and risk of maternal death per 100,000 delivery hospitalizations was also higher in the periviable (98 deaths; aRR 26.4, 95% CI, 16.7–41.9) and preterm periods (31 deaths; aRR 8.7, 95% CI, 6.4–11.7) compared with term (three deaths) (Table 2).

**Table 2.** Severe Maternal Morbidity Incidence and Risk by Gestational Age*

Maternal Morbidity	Periviable (20–25 wk) (n=137,180)	RR (95% CI)	aRR (95% CI)	Preterm (26–36 wk) (n=2,091,010)	RR (95% CI)	aRR (95% CI)	Term (37–42 wk) (n=19,980,299)
SMM	6,225 (4.5)	9.5 (9.0–10.1)	6.8 (6.4–7.3)	60,395 (2.9)	5.9 (5.8–6.1)	3.6 (3.5–3.7)	99,415 (0.5)
pSMM	4,885 (3.6)	8.6 (8.0–9.2)	5.6 (5.2–6.1)	41,760 (2.0)	4.7 (4.6–4.9)	2.4 (2.3–2.5)	86,290 (0.4)
Transfusion SMM	9,965 (7.3)	5.6 (5.3–5.9)	4.2 (3.9–4.4)	102,270 (4.9)	3.7 (3.6–3.7)	2.2 (2.2–2.3)	276,570 (1.4)
SMM							
Live births only†	2,915 (3.6)	8.8 (8.0–9.6)	5.0 (4.6–5.5)	38,695 (1.9)	4.6 (4.4–4.7)	2.3 (2.2–2.3)	85,295 (0.4)
Vaginally delivered live births‡	735 (2.0)	6.6 (5.6–7.9)	5.3 (4.4–6.3)	10,095 (1.0)	3.3 (3.1–3.5)	2.0 (1.9–2.1)	41,400 (0.3)
Live births excluding multiples§	3,470 (4.9)	10.5 (9.7–11.4)	6.7 (6.2–7.4)	51,025 (2.8)	5.9 (5.8–6.1)	3.6 (3.4–3.7)	96,235 (0.5)
Spontaneous live births‖	1,825 (3.0)	6.0 (5.3–6.7)	4.4 (3.8–5.0)	17,120 (1.6)	3.1 (2.9–3.3)	2.2 (2.0–2.4)	6,020 (0.5)
Medically indicated live births¶	1,610 (8.7)	13.1 (11.7–14.8)	8.6 (7.5–9.8)	29,265 (3.5)	5.0 (4.8–5.2)	3.8 (3.6–3.9)	44,300 (0.7)
Live births without maternal comorbidities#	2,355 (3.6)	8.8 (8.0–9.7)	6.5 (5.8–7.2)	36,555 (2.2)	5.3 (5.1–5.4)	3.4 (3.3–3.5)	76,960 (0.4)
Spontaneous live births without maternal comorbidities**	1,190 (2.3)	5.2 (4.5–6.0)	3.8 (3.3–4.5)	11,695 (1.2)	2.7 (2.5–2.9)	1.8 (1.7–2.0)	4,970 (0.5)
Maternal death (per 100,000 delivery hospitalizations)	135 (98.4)	37.1 (24.0–57.4)	26.4 (16.7–41.9)	640 (30.6)	11.5 (8.9–15.0)	8.7 (6.4–11.7)	530 (2.7)

RR, relative risk; aRR, adjusted relative risk; SMM, severe maternal morbidity; pSMM, potentially preventable severe maternal morbidity.

Data are n (%) unless otherwise specified.

* Model includes advanced maternal age, race or ethnicity, Medicaid or Medicare insurance, hospital location and teaching status, chronic psychiatric disease, chronic cardiac disease, chronic hypertension, severe preeclampsia, anemia, twins or higher-order multiples, pregestational diabetes, prior cesarean delivery, and coronavirus disease 2019 (COVID-19). Counts included are weighted from the National Inpatient Sample.

† Analysis limited to individuals with live births only.

‡ Analysis limited to individuals with vaginally delivered live births.

§ Analysis limited to individuals with live births excluding twins and higher-order multiples.

‖ Analysis limited to individuals with spontaneous live births (includes International Classification of Diseases, Tenth Revision, Clinical Modification [ICD-10-CM] codes for preterm labor, preterm prelabor rupture of membranes, and prelabor rupture of membranes; excludes ICD-10-CM diagnosis codes for induction of labor).

¶ Analysis limited to individuals with medically indicated live births (includes ICD-10-CM codes for induction of labor, fetal growth restriction, and preeclampsia).

# Analysis limited to individuals with live births; excludes those with chronic medical conditions (including chronic cardiovascular, respiratory, and renal disease, chronic hypertension, and pregestational diabetes mellitus).

** Analysis limited to individuals with spontaneous live births; excludes those with chronic medical conditions (includes ICD-10-CM codes for preterm labor, preterm prelabor rupture of membranes, and prelabor rupture of membranes, and excludes induction of labor, chronic cardiovascular, respiratory, and renal disease, chronic hypertension, and pregestational diabetes mellitus).

In the sensitivity analysis that included live births only and live births delivered vaginally, the rate and risk of SMM followed a similar pattern as in the primary analysis but to a lesser degree; SMM risk among individuals with live births excluding multifetal gestations was similar to that in the primary analysis (Table 2). When the analysis was limited to individuals with a medical indication for preterm delivery, SMM rate and risk among those with periviable deliveries (8.7%, aRR 8.6, 95% CI, 7.5–9.8) was higher than in the original analysis but similar among individuals with preterm deliveries (3.5%, aRR 3.8, 95% CI, 3.6–3.9). Among live births in individuals without chronic medical conditions, rate and risk of SMM were similar to the original analysis for all delivery age groups; however, when limited to spontaneous births, rate and risk of SMM remained elevated, although to a lesser degree among those with periviable births (2.3%, aRR 3.8, 95% CI, 3.3–4.5) and preterm births (1.2%, aRR 1.8, 95% CI, 1.7–2.0) compared with term births (0.5%) (Table 2).

By organ system classification of SMM, the associated risks of cardiovascular, renal, respiratory, septic, shock, and hemorrhagic SMM events were highest among individuals with periviable deliveries compared with those with preterm and term deliveries (Table 3). However, individuals with preterm births were at significantly higher risk for eclampsia compared with those with periviable and term births (Table 3). Individuals with periviable and preterm deliveries had a threefold to fourfold higher adjusted risk of thromboembolic SMM and hysterectomy compared with those with term deliveries (Table 3).

**Table 3.** Incidence of Individual Indicators of Severe Maternal Morbidity by Gestational Age

Maternal Morbidity*	Periviable (20–25 wk) (n=137,180)	RR (95% CI)	aRR (95% CI)	Preterm (26–36 wk) (n=2,091,010)	RR (95% CI)	aRR (95% CI)	Term (37–42 wk) (n=19,980,299)
Acute myocardial infarction†	80 (5.8)	31.9 (18.7–54.6)	14.4 (8.0–25.5)	400 (1.9)	10.5 (7.6–14.4)	3.9 (2.6–5.9)	365 (0.2)
Aneurysm‡	20 (1.5)	5.5 (2.0–14.8)	3.2 (1.2–8.8)	185 (0.8)	3.3 (2.3–4.9)	2.2 (1.5–3.2)	530 (0.3)
Cardiac arrest or ventricular fibrillation†	115 (8.4)	17.2 (11.2–26.5)	12.5 (8.0–19.5)	675 (3.2)	6.6 (5.3–8.3)	4.6 (3.5–6.0)	975 (0.5)
Conversion of cardiac rhythm†	75 (5.5)	11.3 (6.7–19.0)	7.1 (4.1–12.3)	655 (3.1)	6.5 (5.2–8.1)	4.1 (3.1–5.4)	970 (0.5)
Heart failure or arrest during surgery†	—§	—§	—§	110 (0.5)	10.0 (5.5–18.2)	6.0 (2.7–13.5)	105 (0.1)
Puerperal cerebrovascular disorders	210 (15.3)	7.7 (5.7–10.6)	5.0 (3.6–7.0)	2,385 (11.4)	5.8 (5.1–6.5)	3.4 (3.0–3.9)	3,955 (2.0)
Pulmonary edema or acute heart failure	690 (50.3)	17.7 (14.8–21.0)	5.9 (4.7–7.3)	7,020 (33.6)	11.8 (10.9–12.7)	3.4 (3.1–3.8)	5,715 (2.9)
Total	1,085 (82.2)	14.0 (12.2–16.1)	6.4 (5.5–7.6)	10,595 (51.9)	8.8 (8.3–9.4)	3.4 (3.2–3.7)	11,765 (5.9)
Acute renal failure	1,440 (105.0)	13.6 (12.0–15.5)	5.4 (4.7–6.2)	12,380 (59.2)	7.7 (7.2–8.1)	2.1 (1.9–2.2)	15,535 (7.8)
Adult respiratory distress syndrome	1,145 (83.5)	16.1 (14.0–18.5)	10.1 (8.6–11.8)	10,675 (51.1)	9.8 (9.2–10.4)	5.4 (5.0–5.8)	10,460 (5.2)
Temporary tracheostomy†	100 (7.3)	182.2 (94.5–351.3)	138.4 (70.7–270.9)	250 (1.2)	29.9 (17.2–52.0)	22.1 (12.3–39.9)	80 (0.0)
Ventilation	670 (48.8)	22.8 (19.0–27.4)	14.3 (11.7–17.3)	4,615 (22.1)	10.3 (9.4–11.3)	6.2 (5.5–6.9)	4,295 (2.2)
Severe anesthesia complications‡	15 (1.1)	1.8 (0.6–5.8)	1.7 (0.5–5.3)	230 (1.1)	9.2 (8.7–9.7)	1.7 (1.2–2.4)	1,185 (0.6)
Total	1,270 (96.1)	15.4 (13.5–17.6)	10.1 (8.8–11.7)	11,720 (57.4)	9.2 (8.7–9.7)	5.2 (4.8–5.6)	12,500 (6.3)
Eclampsia	310 (22.6)	4.9 (3.8–6.3)	4.0 (3.0–5.2)	7,500 (35.9)	7.8 (7.2–8.3)	7.1 (6.6–7.6)	9,255 (4.6)
Sepsis	1,805 (131.6)	20.6 (18.3–23.1)	16.9 (15.0–19.2)	5,870 (28.1)	4.3 (4.0–4.7)	3.5 (3.2–3.9)	12,945 (6.5)
Shock	540 (39.4)	9.3 (7.7–11.3)	6.7 (5.4–8.2)	3,595 (17.2)	4.1 (3.7–4.4)	2.6 (2.3–2.9)	8,480 (4.2)
Blood transfusion	5,210 (379.8)	4.0 (3.8–4.3)	3.0 (2.8–3.2)	54,675 (261.5)	2.7 (2.7–2.8)	1.7 (1.6–1.8)	194,600 (97.4)
Disseminated intravascular coagulation	1,395 (101.7)	6.3 (5.6–7.1)	5.1 (4.5–5.8)	11,085 (53.0)	3.3 (3.1–3.5)	2.2 (2.1–2.4)	32,475 (16.3)
Total	6,060 (454.7)	4.2 (4.0–4.5)	3.2 (3.0–3.5)	61,925 (302.0)	2.8 (2.7–2.8)	1.7 (1.7–1.8)	221,160 (111.0)
Air and thrombotic embolism	175 (12.8)	6.0 (4.3–8.5)	4.5 (3.1–6.3)	1,795 (8.6)	4.1 (3.6–4.6)	2.9 (2.5–3.4)	4,220 (2.1)
Sickle cell disease with crisis†	65 (4.7)	9.2 (5.2–16.0)	7.6 (4.3–13.3)	1,250 (6.0)	11.5 (9.6–13.9)	8.5 (6.9–10.5)	1,035 (0.5)
Amniotic fluid embolism‡	15 (1.1)	2.7 (0.9–8.4)	2.4 (0.8–7.6)	205 (1.0)	2.4 (1.7–3.4)	2.2 (1.6–3.1)	815 (0.4)
Total	250 (19.1)	6.3 (4.7–8.3)	4.4 (3.3–5.8)	3,195 (15.7)	5.2 (4.7–5.7)	3.6 (3.2–4.0)	6,030 (3.0)
Hysterectomy||	780 (56.9)	9.6 (8.2–11.4)	4.0 (3.2–5.1)	12,640 (60.5)	10.2 (9.7–10.9)	3.3 (3.1–3.6)	11,850 (5.9)

RR, relative risk; aRR, adjusted relative risk.

Data are n (per 10,000 delivery hospitalizations) unless otherwise specified.

* Model includes advanced maternal age, race or ethnicity, Medicaid or Medicare insurance, hospital location and teaching status, chronic psychiatric disease, chronic cardiac disease, chronic hypertension, severe preeclampsia, anemia, twins or higher-order multiples, pregestational diabetes, prior cesarean delivery, and coronavirus disease 2019 (COVID-19). Counts included are weighted from the National Inpatient Sample.

† Model limited to chronic cardiac disease, prior cesarean delivery, severe preeclampsia, and COVID-19 due to infrequent events.

‡ Model includes chronic cardiac disease due to infrequent events.

§ Unable to be estimated due to rarity of events.

‖ Model additionally includes placenta accreta spectrum.

**Fig. 2.**
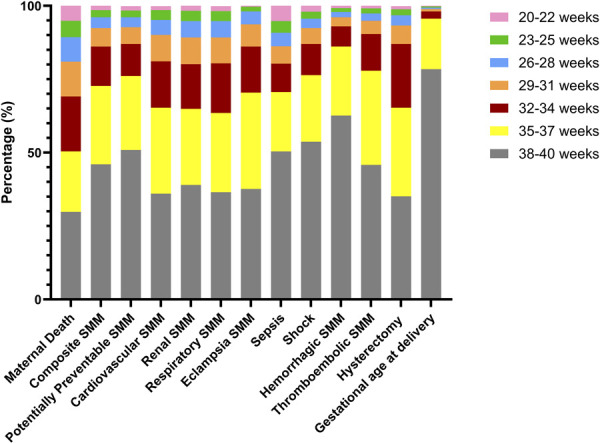
Percentage of severe maternal morbidity or mortality events by 3-week gestational age group (weeks). Total population proportion by gestational age on the far right as comparison. Cardiovascular severe maternal morbidity (SMM): acute myocardial infarction, aneurysm; cardiac arrest or ventricular fibrillation, conversion of cardiac rhythm, puerperal cerebrovascular disorders, and pulmonary edema or acute heart failure. Respiratory SMM: adult respiratory distress syndrome, severe anesthesia complication, temporary tracheostomy, and ventilation. Hemorrhage SMM: transfusion and disseminated intravascular coagulation. Thromboembolic SMM: amniotic fluid embolism, sickle cell disease with crisis, and air and thrombotic embolism.

Rates of SMM by organ system category varied across 3-week gestational age groups (Appendices 3 and 4, available online at http://links.lww.com/AOG/E152). Individuals with deliveries between 20 and 22 weeks of gestation had the highest rate of sepsis-related SMM. There was a crescendo of increasing rates of overall SMM and cardiovascular, renal, respiratory, and eclampsia-related SMM, with the highest incidence among individuals with deliveries from 28 to 33 weeks of gestation. The rate of hysterectomy peaked between 32 and 34 weeks of gestation. The rate of maternal death was significantly increased (88–150 deaths/100,000 delivery hospitalization) for patients who delivered before 32 weeks of gestation (Appendices 3 and 4, http://links.lww.com/AOG/E152). Although deliveries occurring before 37 weeks of gestation represent approximately 10% of the study population, these individuals had a disproportionate burden of SMM and mortality (Fig. 2). Additionally, individuals delivering before 35 weeks of gestation comprised approximately 50% of maternal deaths (Appendix 3, http://links.lww.com/AOG/E152, and Fig. 2).

*Czarny. SMM With Periviable and Preterm Birth. O&G Open 2025*.

**Fig. 3.**
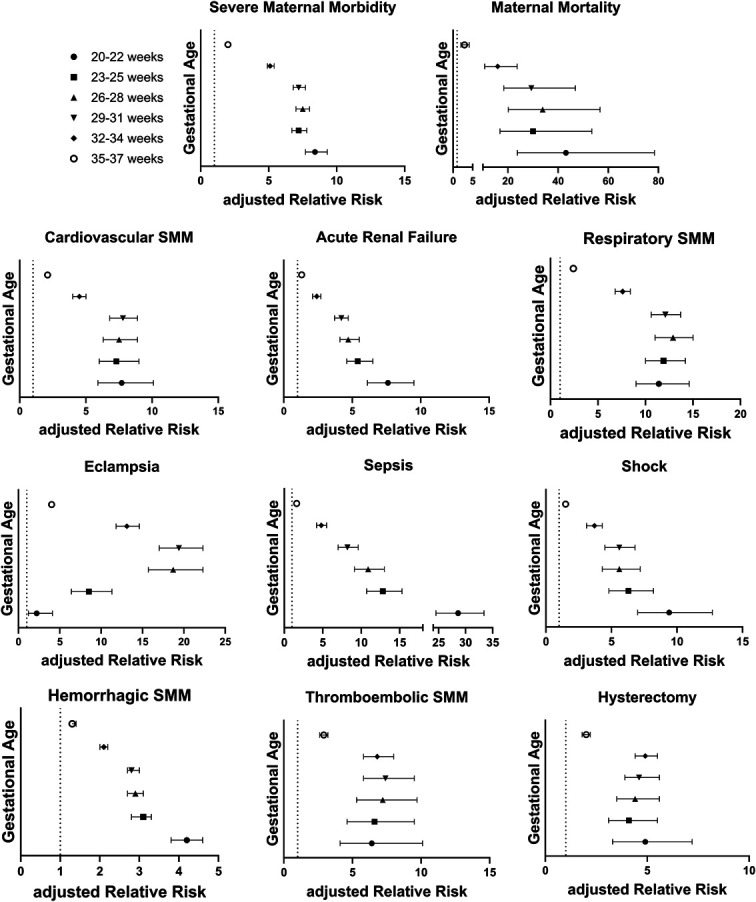
Risk of severe maternal morbidity (SMM). Data are adjusted relative risk with 95% CI. Cardiovascular SMM: acute myocardial infarction, aneurysm, cardiac arrest or ventricular fibrillation, conversion of cardiac rhythm, puerperal cerebrovascular disorders, and pulmonary edema or acute heart failure. Respiratory SMM: adult respiratory distress syndrome, severe anesthesia complication, temporary tracheostomy, and ventilation. Hemorrhage SMM: transfusion and disseminated intravascular coagulation. Thromboembolic SMM: amniotic fluid embolism, sickle cell disease with crisis, and air and thrombotic embolism. Main model includes advanced maternal age, race and ethnicity, government insurance, hospital location and teaching status, chronic psychiatric disease, chronic cardiac disease, chronic hypertension, severe preeclampsia, anemia, twins or higher-order multiple gestation, pregestational diabetes, prior cesarean delivery, and coronavirus disease 2019 (COVID-19). Model for maternal mortality and thromboembolic SMM includes chronic cardiac disease, previous cesarean delivery, severe preeclampsia, and COVID-19 due to infrequent events. Model for eclampsia adjusted for chronic cardiac disease, previous cesarean delivery, and COVID-19 due to infrequent events. Model for hysterectomy additionally includes placenta accreta spectrum.

The risk of SMM was sevenfold to eightfold higher at gestational ages of less than 32 weeks, and risk remained significantly increased for individuals delivering between 32–34 weeks and 35–37 weeks relative to the 38–40-week cohort, but to a lesser extent. Sensitivity analyses limited to individuals with spontaneous live births without chronic medical conditions found similar SMM risk associated with 3-week gestational age cohorts as the primary analysis (Appendix 5, available online at http://links.lww.com/AOG/E152). A higher risk of maternal death was associated with delivery before 35 weeks of gestation (Appendix 5, http://links.lww.com/AOG/E152, and Fig. 3).

*Czarny. SMM With Periviable and Preterm Birth. O&G Open 2025*.

Risks of all SMM categories were significantly increased with delivery before 38 weeks of gestation (Appendix 5, http://links.lww.com/AOG/E152, and Fig. 3). The risk of sepsis and adverse pregnancy outcomes was highest among individuals who delivered between 20 and 22 weeks of gestation, and eclampsia was highest among those delivered between 26 and 31 weeks. The risks of thromboembolic SMM and hysterectomy were highest at gestational ages less than 34 weeks, with decreasing risk with higher gestational age at delivery (Appendix 5, http://links.lww.com/AOG/E152, and Fig. 3).

The aPAF between the outcome of SMM and exposure of periviable birth (4.7%, 95% CI, 4.4–5.0%) was lower compared with the aPAF between SMM and preterm birth (26.3%, 95% 25.6–27.1%) (Appendix 6, available online at http://links.lww.com/AOG/E152). Likewise, the aPAF between the outcome of maternal death and exposure of periviable birth (19.7%, 95% CI, 12.1–26.7%) also was lower compared with the aPAF between SMM and preterm birth (48.1%, 95% 39.9–55.2%). Sensitivity analyses limited to individuals with live births without chronic medical conditions indicated a lower aPAF of SMM compared with the original analysis. However, the aPAF of SMM was higher for both periviable and preterm deliveries among individuals with live births without chronic medical conditions and spontaneous labor compared with the original analysis (Appendix 6, http://links.lww.com/AOG/E152).

## DISCUSSION

In this population-based retrospective cohort study, deliveries occurring before 37 weeks of gestation were associated with a higher risk of SMM and maternal mortality, with the highest risk among individuals delivering in the periviable period. Individuals delivering before 37 weeks of gestation had a disproportionate rate of SMM and maternal death burden than those delivering at term. Although the risk for category-specific SMM outcomes had some variability among 3-week gestational age groupings, gestational ages less than 35 weeks were associated with increased risk across nearly all categories.

The results remained consistent across various groupings, suggesting that the increased risk of SMM is not simply a byproduct of conditions such as hypertension, diabetes, or other preexisting maternal conditions, but rather is a broader consequence of delivering at preterm or periviable gestations. Additionally, the aPAF for SMM among individuals with periviable and preterm births remained and was significantly greater among those with spontaneous birth without medical comorbidities, further highlighting the effects of preterm and periviable birth on SMM. This finding is particularly significant because it challenges the conventional focus on maternal comorbidities as a primary risk factor for SMM. Although maternal health certainly plays a role in outcomes, the gestational age at delivery appears to be an independent risk factor in itself.

Regionalization of care for those at risk for preterm delivery has long been established as a way of improving outcomes of preterm neonates.^34,35^ Risk-appropriate regionalization of care for pregnant individuals with high-risk conditions was introduced by the American College of Obstetricians and Gynecologists and the Society for Maternal-Fetal Medicine in response to the rising rate of SMM and mortality in the U.S.^34^ Since its implementation in 2015, regionalization of maternal care has decreased the risk of severe adverse outcomes for selected risk conditions.^36,37^

Despite the benefits of regionalization of perinatal care, risk-appropriate neonatal care does not always align with risk-appropriate maternal care, or vice versa. A spatial and proximity analysis of obstetric and neonatal critical care units, found that 18% of level III obstetric care units and 20% of level III or IV neonatal care units did not have a complimentary neonatal or maternal care unit within a 10-mile radius.^38^ These potential gaps in coordinated care are further highlighted by findings from Easter et al,^39^ who report that, although only 2.41% of deliveries occurred at hospitals with an inappropriate level of maternal care, 43.4% of individuals with high-risk conditions requiring level III or IV care delivered at hospitals with a lower level of maternal care than recommended. Importantly, the data presented here highlight that there is a strong association between SMM and preterm and periviable birth, even among individuals with spontaneous deliveries without concurrent high-risk medical comorbidities. In situations involving threatened preterm or periviable labor, the level of risk-appropriate maternal care should be given equal priority to that of neonatal care to ensure comprehensive care. Without integrated systems for risk-appropriate care, delays in treatment or fragmented care for the mother–child dyad are likely, potentially compromising outcomes for both. Lack of coordination between neonatal and maternal care facilities remains a barrier to effective regionalization and addressing this discordance is essential for optimizing perinatal care.

The results of this study must be taken in the context of several limitations, including the possibility of missing data and misclassification, underestimation, or overestimation of exposures and outcomes inherent in the use of ICD codes.^40^ Further, exclusion of individuals transferred to acute care hospitals may add bias to these results. Additionally, no conclusions can be drawn regarding the effect of regionalization of care on SMM from this study. This analysis includes only SMM events occurring during delivery hospitalization; therefore, inferences regarding SMM events outside of delivery hospitalization cannot be drawn. Although an association between preterm birth or periviable birth and SMM exists, determining causality is challenging. For instance, sepsis may lead to preterm labor, or tocolytic treatment for threatened preterm birth might result in sepsis from an intra-amniotic infection. In such cases, preterm birth and maternal complications may mutually influence each other, making it difficult to establish whether preterm birth directly causes SMM or if underlying conditions exacerbate both simultaneously, complicating the interpretation and direction of causality.

Despite these limitations, the use of a large national database allows for analysis of a generalizable and contemporary sample that is representative of the U.S. population. Moreover, the robust cohort allows for risk stratification by 3-week gestational age group and insight into occurrence of infrequent SMM events. Furthermore, the ability to adjust for multiple comorbidities and baseline characteristics and the use of sensitivity analyses allows for improved credibility of our results.

We found a high occurrence and risk of SMM and mortality among individuals who deliver in the periviable and preterm period. These data support the need for coordination of maternal and neonatal risk-appropriate care to improve outcomes for the maternal–child dyad.
